# Rapid identification and deployment of major genes for flowering time and awn traits in common wheat

**DOI:** 10.3389/fpls.2022.992811

**Published:** 2022-08-26

**Authors:** Jizhong Wu, Linyi Qiao, Ying Liu, Bisheng Fu, Ragupathi Nagarajan, Yahya Rauf, Haiyan Jia, Liuling Yan

**Affiliations:** ^1^Institute of Germplasm Resources and Biotechnology, Jiangsu Academy of Agricultural Sciences, Nanjing, Jiangsu, China; ^2^Department of Plant and Soil Sciences, Oklahoma State University, Stillwater, OK, United States; ^3^College of Agronomy, Shanxi Key Laboratory of Crop Genetics and Molecular Improvement, Shanxi Agricultural University, Taiyuan, Shanxi, China; ^4^The Applied Plant Genomics Laboratory, National Key Laboratory of Crop Genetics and Germplasm Enhancement, Nanjing Agricultural University, Nanjing, Jiangsu, China

**Keywords:** *Vrn-D1*, flowering genes, *Ali-A1*, awn, common wheat

## Abstract

Molecular markers are developed to accelerate deployment of genes for desirable traits segregated in a bi-parental population of recombinant inbred lines (RILs) or doubled haplotype (DH) lines for mapping. However, it would be the most effective if such markers for multiple traits could be identified in an F_2_ population. In this study, single nucleotide polymorphisms (SNP) chips were used to identify major genes for heading date and awn in an F_2_ population without developing RILs or DH lines. The population was generated from a cross between a locally adapted spring wheat cultivar “Ningmaizi119” and a winter wheat cultivar “Tabasco” with a diverse genetic background. It was found that the dominant *Vrn-D1* allele could make Ningmaizi119 flowered a few months earlier than Tabasco in the greenhouse and without vernalization. The observed effects of the allele were validated in F_3_ populations. It was also found that the dominant *Ali-A1* allele for awnless trait in Tabasco or the recessive *ali-A1* allele for awn trait in Ningmaizi119 was segregated in the F_2_ population. The allelic variation in the *ALI-A1* gene relies not only on the DNA polymorphisms in the promoter but also on gene copy number, with one copy *ali-A1* in Ningmaizi119 but two copies *Ali-A1* in Tabasco based on RT-PCR results. According to wheat genome sequences, cultivar “Mattis” has two copies *Ali-A1* and cultivar “Spelta” has four copies *Ali-A* in a chromosome that was uncharacterized (ChrUN), in addition to one copy on chromosome 5A. This study rapidly characterized the effects of the dominant *Vrn-D1* allele and identified the haplotype of *Ali-A1* in gene copy number in the F_2_ segregation population of common wheat will accelerate their deployment in cycling lines in breeding.

## Introduction

Wheat (*Triticum aestivum* L. 2*n* = 6*x* = 42, AABBDD) is the largest crop in production and acreage in China, where 10 major agroecological zones are classified on the basis of differences in growth habit types and responses of cultivars to low temperature and photoperiod (Zhuang, 2003). Zone III is the Middle and Lower Yangtze Valley Zone, where it occupies approximate 15% of total production and acreage in China and all wheat cultivars are sown in autumn and harvested in next summer (Guo et al., 2015). Wheat cultivars adapted to this Zone include 53% winter type, 36% spring type, and 11% cultivars, whose genotypes are unknown based on the vernalization genes (Zhang et al., 2008). The co-existence of spring and winter wheat cultivars make it confused with cultivar classification, resulting in calls as strong spring, semi-spring, and semi-winter type, etc. (Jin, 1997; Dong and Zheng, 2000; Zhuang, 2003). As a result, spring wheat cultivars are sometimes planted in the northern part of Zone III, resulting in frozen damage in the spring. Conversely, winter wheat cultivars are sometimes grown in the southern region of Zone III, resulting in delayed development but earlier ripe due to high temperature in the summer. Therefore, precise identification of genes that make new varieties adapted to local environments is crucially important in breeding programs.

The adaptability of wheat cultivars is largely governed by genes in three pathways, including vernalization response, photoperiod response (*PPD*), and earliness *per se* (*EPS*; Kato and Yamagata, 1988; Laurie et al., 1995; Snape et al., 2001). Among them, vernalization is the most effective mechanism underling wheat flowering and hence the basic adaptation of a genotype for a particular environmental condition (Flood and Halloran, 1986; Griffiths et al., 2003). Major genes responsible for the difference in vernalization requirement between the two distinct types have been cloned, including *VRN-1* (Yan et al., 2003), *VRN-2* (Yan et al., 2004b), *VRN-3* (Yan et al., 2006), and *VRN-D4* (Kippes et al., 2015) that are promoted by low temperature, as well as *VRN-2* (Yan et al., 2004b) that is repressed by low temperature. When the regulatory elements are found in the promoter or intron in one of three homoeologous *Vrn-A1*, *Vrn-B1*, or *Vrn-D1* genes, single nucleotide polymorphism (SNP) is also found in coding region that offers alternative mechanism for flowering time in winter wheat at the protein level. Due to an alternation of an amino acid, “Jagger” has the vrn-A1a protein for weak winter wheat or less vernalization requirement, which is dominant to the stronger winter wheat requiring more vernalization to reach a vernalization saturation point in cultivar “2174” that has the vrn-A1b protein (Li et al., 2013). Major genes responsible for the difference in photoperiod sensitivity are three homoeologous *PPD* genes that are isolated according to the sequence of the barley *PPD-H1* gene (Turner et al., 2005; Beales et al., 2007).

“Tabasco” was a wheat cultivar released in Germany. This cultivar was introduced to Zone III, owing to the presence of a new powdery mildew resistant gene *Pm48* conferring outstanding resistance against powdery mildew and ideal spike architecture (Gao et al., 2012). Tabasco should be readily easy resistance source in breeding, but it is a strong winter cultivar that is mainly adapted to areas with average January temperature between −7 and 4°C to meet the strong requirement for vernalization (Iwaki et al., 2000).

Tabasco is awnless that is a characteristic of European cultivars, whereas most cultivars in Zone III have awn. It was reported that a gene encoding a C2H2 zinc finger and ethylene-responsive element binding factor-associated amphiphilic repression (EAR) motifs plays as Awn Length inhibitor 1 (*ALI1*) that suppresses downstream genes transcriptionally resulting in the awnless trait (DeWitt et al., 2020; Huang et al., 2020; Niu et al., 2020; Wang et al., 2020; Würschum et al., 2020). Although *ALI1* was identified in these independent studies, allelic variations or sequence polymorphisms between the *Ali1* allele for awnless and the *ali-1* allele for awn are inconsistent in reported genetic materials or germplasm. When Tabasco with the awnless trait was introduced into another cultivar with distant genetic backgrounds, it could be difficult in selection for the homozygous allele for the awnless or awn trait in early generations.

Molecular markers can be developed to accelerate deployment of genes for the desirable traits by using two parents with contrasting phenotypes for a cross to generate a biparental population of recombinant inbred lines (RILs) or doubled haplotype (DH) lines for mapping. However, it would be the most effective if multiple traits could be mapped in an F_2_ population. SNP chips have provided scientists with unprecedented tools to unravel allelic variation associated with complex traits (Poland and Rife, 2012). In this study, we used SNP technology to identify markers across an entire genome for genotyping of an F_2_ population. The segregating population has provided a successful example of developing high-density markers in wheat without developing RILs or DH lines.

## Materials and methods

### Plant materials

Tabasco (pedigree: ZE-90-2666/86-Z-99-9//CPB-93-27) was provided by Dr. Andreas Jacobi. Tabasco was crossed with Ningmaizi119 (abbreviated Zi119 hereafter), which is a hard red spring wheat cultivar (pedigree: Su13577/Ningmai13//Ningmai13) and released by the Crop Variety Approval Committee of Jiangsu Province. The two parental lines were vernalized at 4°C with long day photoperiod for 6 weeks to determine their growth habit. Five hybrid seeds were produced and self-pollenated to generate a population of 212 F_2_ plants. The F_2_ population was tested in a greenhouse conditioned with 16 h for light at 25 ± 2°C, and 8 h for darkness at 20 ± 2°C during the whole life cycle, and the greenhouse is located on Stillwater campus of Oklahoma State University. In addition to natural sunlight, high-pressure sodium lamps were used to provide supplemental lighting for nights and raining days. All of the population plants were not vernalized to identify genes associated with early flowering genes without requirement of vernalization. Since the F_2_ population was tested, no replicate could be conducted. No experiment was conducted to test how environmental cues affect the quantitative trait loci (QTLs) found in this study. The F_2_ population consisted of 212 plants that could have no replicates for a specific genotype. The heading date of a plant was recorded when the first spike of the plant was emerging from the flag leaf sheath of the main stem or a primary tiller. Heading date was recorded for each plant of the population when the main stem of the plant headed up. Flowering time was recorded for each plant of the population when the spike on the main stem of the plant flowered.

### Genotypes of Tabasco × Zi119 F_2_ population

Genomic DNA was extracted from parental lines and the 212 F_2_ individual plants using the method as described in Dubcovsky et al. (1994). PCR markers for genes known to regulate flowering time were used to identify polymorphism between Tabasco and Zi119.

A 55 k SNP chip was used to genotype 105 F_2_ plants and two parental lines at Golden Marker Biotech Co., Ltd., Beijing, China, and the number of 107 samples from this project was used to match up three plates with other projects. The 105 plants were randomly selected at seedling stages without knowing growth habit. A total of 4,657 markers was analyzed using IciMapping v4.1, and 3,459 of them were developed after excessive missing data were removed in the BIN program. The 3,459 SNP markers were assembled into 21 chromosome linkage groups forming genetic maps for the F_2_ population (Supplementary Table S1), based on a Chinese Spring genome sequence in the International Wheat Genome Sequencing Consortium (IWGSC) RefSeq v1.1.^1^ The 3,459 markers were integrated with the phenotypes of the 105 F_2_ plants to screen QTL in the BIP procedure, with 2.5 value of the threshold.

### Allelic variation and gene expression of *VRN-D1*

Two pairs of primers were used to test polymorphisms in *VRN-D1* between Tabasco and Zi119. The first primer pair were VRN-D1F (5′-GTTGTCTGCCTCATCAAATCC-3′) and VRN-D1R3 (5′-GGTCACTGGTGGTCTGTGC-3′) that were used to detect a deletion in intron one of *VRN-D1*, and the second primer pair were VRN-D1F (5′-GTTGTCTGCCTCATCAAATCC-3′) and VRN-D1R4 (5′-AAATGAAAAGGAACGAGAGCG-3′) that were used to detect an insertion in intron one of *VRN-D1* (Fu et al., 2005). PCR amplification conditions were: 94°C for 3 min, 40 cycles of 94°C for 45 s, 58°C for 45 s, and then 72°C for 4 min, with a final extension of 72°C for 10 min. qRT-PCR was used to determine the transcriptional levels of *VRN-D1* in two parental lines at seedling state without vernalization. RNA samples were collected from leaves, and total RNA was extracted using TRIzol® reagent (Invitrogen, Carlsbard, CA, United States). cDNA was synthesized from 5 μg of RNA using SuperScript™ II Reverse Transcriptase Kit (Invitrogen, Carlsbard, CA, United States). *Primers for VRN-D1* transcripts were VRN1D-RT-F2 (5′-ATGCTCCCCCTGCCGCAG-3′) and VRN1D-RT-R2 (5′-GCTGCACTGCCGCATCCC-3′).

### Gene expression and copy number variation of *ALI1*

Awn Length inhibitor 1 was isolated from gDNA using specific primers ALI1-F1 (5′-CCATGTCTGTGGGCTCTGTT-3′) and ALI-R1 (5′-GCCTATAGGACTAGCCCATATAC-3′). The amplified PCR products were directly sequenced to identify allelic variation in the sequenced region between the two parental lines. RT-ALI-F1 (GTTCGCCTGCTCCTACTGCT) RT-ALI-R1 (GTGGTTCTCGATGGCGAGCT) were used to determine the transcription levels of ALI1 using cDNA as template and actin as an endogenous control, and the same primers determine the copy number of *ALI1* using gDNA as template and *TaCO2* as an endogenous control for one copy gene on the entire genome (Li et al., 2013).

## Results

### Segregation of developmental phases in Tabasco × Zi119 F_2_ population

**Figure 1:**
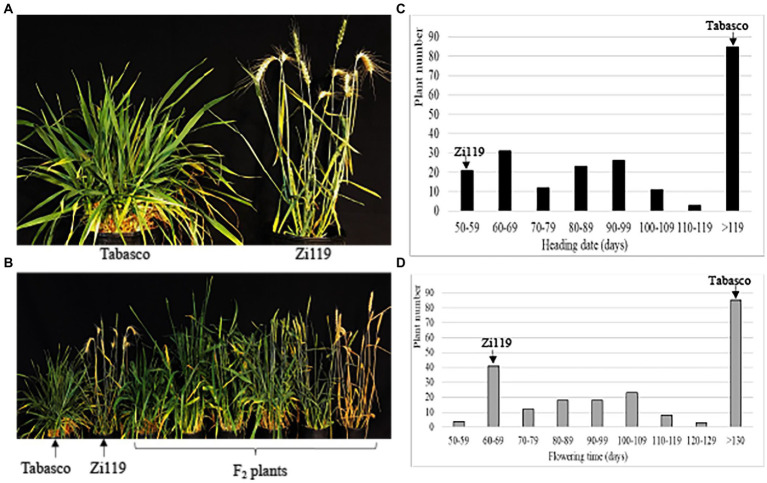
Difference in developmental phases between Tabasco and Zi119. **(A)** Comparison of heading date between Tabasco and Zi19. **(B)** Segregation of phenotypes in populations. **(C)** Frequency distribution of heading date for the F_2_ population. **(D)** Frequency distribution of flowering time for the F_2_ population. **(C,D)** Arrows indicate the groups the two parent lines fell into based on their phenotypes. The AX plus eight-digit single nucleotide polymorphism (SNP) codes are markers from SNP chips.

When tested under long-day and without vernalization across the experiment, Zi119 headed 50 days after planting, whereas Tabasco did not head up 150 days after planting before it was vernalized (Figure 1A). Without vernalization, Zi119 plants flowered; hence, this cultivar was designated as “spring” wheat. Without vernalization, Tabasco did not flower within 5 months; but with 6 weeks’ vernalization at 4°C and long day conditions, it flowered 120 days; hence, this cultivar was designated as “winter” wheat. Tabasco was also a strong winter wheat, compared with a weak winter wheat cultivar “Jagger” that headed 92 days without vernalization and was accelerated 30 days for heading by 3 weeks’ vernalization (Li et al., 2013). F_1_ plants from Tabasco × Zi119 headed 75 days after planting, indicating that those genes for spring growth habit in Zi119 were primarily dominant to those for winter growth habit in Tabasco.

The 212 F_2_ plants showed a large segregation in heading date and flowering time (Figure 1B). Without vernalization, the Tabasco × Zi119 F_2_ plants showed clear segregation developmental processing but did not show a clear cut-off for growth habit to distinguish between spring wheat and winter wheat. Instead, the heading date of the 127 F_2_ plants was continuous within 120 days after planting, whereas the heading date of the remaining 85 F_2_ plants was extremely late. Plant number distribution of the 212 F_2_ plants for heading date was shown in Figure 1C, indicating that a major gene for spring habit might be present in the population and minor genes might have modified the heading date. Plant number distribution of the 212 F_2_ plants showed a similar pattern in flowering time (Figure 1D), indicating that no gene was segregated for developmental phase from heading to flowering in the population.

### Genotypes of the Tabasco × Zi119 F_2_ population using SNP chips

A total of 53,063 SNP calls were generated for 105 F_2_ plants, and 9,023 (17%) of these calls showed allelic variation between the parental lines. From these 9,023 polymorphic calls, 4,657 markers were developed, according to two criteria. One is that one marker had less than 8% missing data in the 105 plants in the population, and the other is that one marker had a segregation ratio of 1: 2: 1 for A: H: B alleles (Chi-Square Test, *p* < 0.27), with the homozygous A allele for Tabasco, the homologous B allele for Zi119, and H for the heterozygous allele. Detailed information for the length of each linkage group and genetic distances of the SNP markers on the whole genome is provided in Supplementary Table S1.

The 3,459 SNP markers that were mapped in the F_2_ population spanned 8,118 cM, with 0.43 cM per marker in genetic distance. The 3,459 markers were integrated with the phenotypes of the 105 F_2_ plants to screen QTL and identified genomic regions that had significant effects on two traits segregated in the Tabasco × Zi119 F_2_ population. The results suggested that the SNP chips could be used to rapidly genotype an F_2_ population for mapping of QTL for the important traits.

### Rapid mapping of QTLs for heading date and flowering time in the Tabasco × Zi119 F_2_ population

**Figure 2:**
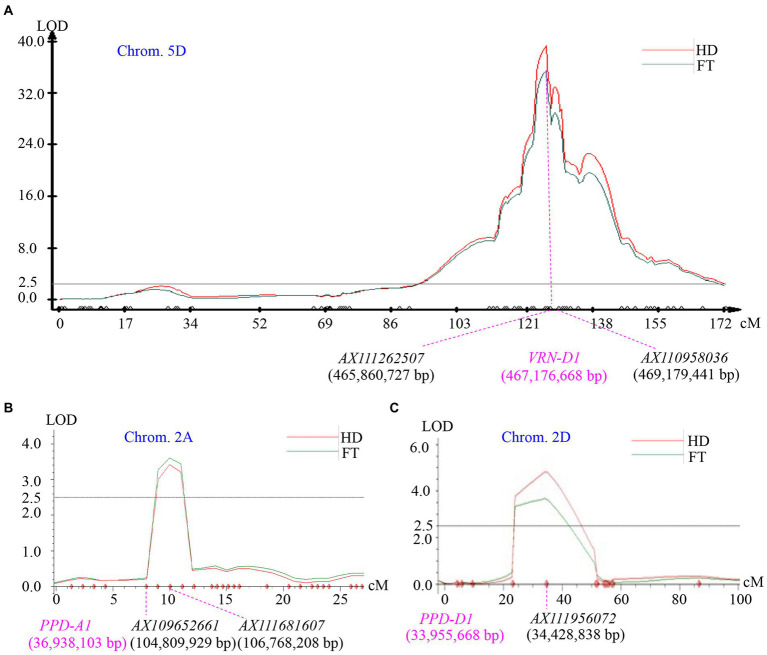
Mapping of quantitative trait loci (QTLs) for heading date segregated in the Tabasco and Zi119 population. **(A)** A major QTL on chromosome 5D. The peak of this QTL is associated with the *VRN-D1* gene. **(B)** A minor QTL on chromosome 2A. The peak of this QTL is not associated with the *PPD-A1* gene. **(C)** A minor QTL on chromosome 2D. The peak of this QTL is associated with the *PPD-D1* gene. The phenotypic data was for heading date (HD) or flowering time (FT). LOD value is indicated on Y axis, and genetic distance (cM) is indicated on *X* axis. The physical locations of the genes are indicated in pink based on the Chinese Spring genome sequence of RefSeq v1.1.

A major QTL for heading date and flowering time was simultaneously mapped to chromosome 5D, where a total of 81 SNP markers was assembled a linkage group (Figure 2A). The LOD value at the peak position of the QTL heading date and flowering time was 39.1 and 38.3 respectively, explaining 78.3 and 79.2% of the total phenotypic variation. Based on the genotypes of *AX110958036* at the mapped QTL locus in the 105 F_2_ plants, plants homozygous for the Zi119 allele headed at averaged 62.3 ± 1.8 (*n* = 24), which was significantly earlier than 189.9 ± 7.0 days (*n* = 22) for plants carrying the homozygous Tabasco allele (*p* = 4.66E−21). Plants that had heterozygotes averaged 110.6 ± 4.2 days (*n* = 59) also headed significantly earlier than plants carrying the homozygous Tabasco allele (*p* = 9.93E−10). In addition, the plants homozygous for the Zi119 allele headed significantly earlier than the plants that had heterozygotes (*p* = 1.23E−15). Collectively, the Zi119 allele was partially dominant for early heading over the Tabasco allele for late heading.

Two additional minor QTLs for heading date and flowering time were detected using the same phenotypic data from the Tabasco × Zi119 F_2_ population. One was on chromosome 2A with LOD value 3.4 and explained 1.8% of the total variation for heading date, and with LOD value 3.6 and explained 2.1% of the total variation for flowering time (Figure 2B). The other one was on chromosome 2D with LOD value 4.8 and explained 2.8% of the total variation for heading date, and with LOD value 3.7 and explained 2.2% of the total variation for flowering time (Figure 2C). No gene for heading date and flowering time was reported on the minor QTL locus on chromosome 2A, but it was likely that *PPD-D1* could be the gene causing the minor QTL on chromosome 2D (Figure 2C). The Zi119 allele might have an allele for insensitivity to photoperiod for early heading (101.9 ± 4.6, *n* = 21), whereas Tabasco an allele for sensitivity to photoperiod for late heading (132.4 ± 5.0, *n* = 29).

To accelerate utilizing the gene at the major QTL on chromosome 5D in breeding, this QTL was validated using F_3_ progeny plants and the candidate gene causing the QTL was identified.

### Validation of the QTLs in F_3_ generations

**Figure 3:**
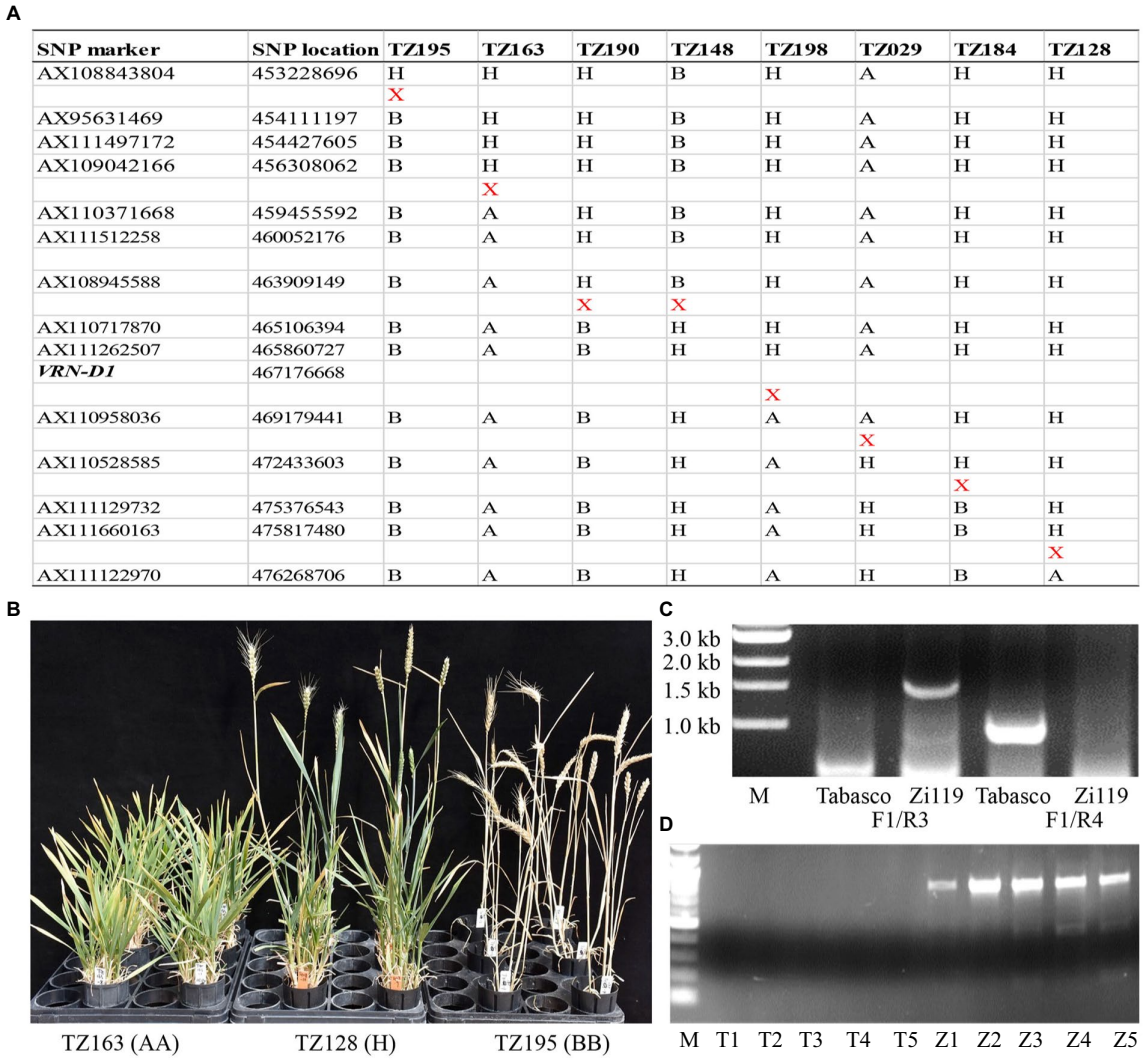
Genotypes and phenotypes of recombinant plants that have crossovers at the *VRN-D1* locus. **(A)** Physical locations of eight crossovers that occurred at the *VRN-D1* locus. “*X*” indicates a crossover detected between two neighboring markers. **(B)** Images for developmental processes in three recombinant lines in their respective F_3_ populations. **(C)** The PCR marker for InDel between the *Vrn-D1* allele in Zi119 and the *vrn-D1* allele in Tabasco. **(D)** PCR products from cDNA samples from each of five random plants carrying the *Vrn-D1* allele from Zi119 (Z1–Z5) and the *vrn-D1* allele from Tabasco (T1–T5).

Eight F_2_ plants that had crossovers in the QTL region (Figure 3A) were selected to generate F_3_ progeny families for testing in the same greenhouse. These F_3_ progeny plants were not vernalized throughout the experiment. All plants in two families that had the Zi119 allele at marker *AX111262507* headed very earlier, 71 days in TZ195 and 74 days in TZ190. All plants in two families (TZ163 and TZ029) that had the Tabasco allele at marker *AX111262507* did not head up until 150 days after planting when the experiment was terminated. Plants in the other four families (TZ148, TZ198, TZ184, and TZ128) that had heterozygotes showed segregation in heading date. Images for three families with different genotypes of *AX111262507* were taken (Figure 3B). These results validated the major QTL on chromosome 5D for heading date and flowering time. Moreover, the gene causing the major QTL on chromosome 5D was linked with *AX111262507*.

### Allelic variation in *VRN-D1* associated with the major QTL

*VRN-D1* on chromosome 5D (*TraesCS5D02G401500*) was positioned at 467,176,668 bp, which is close to the linked marker *AX111262507* at 465,860,727 bp (Figure 3A); therefore, *VRN-D1* naturally and reasonably became a candidate gene for the mapped QTL for heading date and flowering time. Two pairs of primers that are specific to *VRN-D1* (Fu et al., 2005) were used to distinguish between the presence and absence of a 4,235-bp deletion in intron one in Tabasco and Zi119. The first primer pair, VRN-D1F and VRN-D1R3 that were designed to produce an amplification product only when the deletion was present (i.e., the spring allele or dominant *Vrn-D1* allele), showed a 1,671-bp PCR product from Zi119 but not from Tabasco, demonstrating the 4,235-bp deletion was present in Zi119 but not in Tabasco (Figure 3C). The second primer pair, VRN-D1F and VRN-D1R4 that were designed to produce an amplification product only when the deletion was absent (i.e., the winter allele or dominant *vrn-D1* allele), showed a 997-bp PCR product from Tabasco but not from Zi119, demonstrating the 4,235-bp insertion was present in Tabasco but not in Zi119 (Figure 3C). The amplified PCR products were directly sequenced, and the sequences showed 100% identity to the *VRN-D1* InDel. Besides this InDel, no other differences were found in the *VRN-D1* coding region between the two parental lines. Furthermore, *Vrn-D1* was expressed in Zi119 at its seedling stage, but *vrn-D1* was not expressed in Tabasco at its seedling stage (Figure 3D). The diagnostic marker for the InDel variation in *VRN-D1* was used to genotype the F_3_ progeny families. Results showed that the plants that had homozygous and heterozygous deletion in the *Vrn-D1* allele headed up earlier whereas the plants that had homozygous insertion in the *vrn-D1* allele headed up later. These results supported that Zi119 carried the dominant *Vrn-D1* allele that enabled the plant to head up as early as 70 days after planting and without vernalization.

### The genome region associated with the presence and absence of awn

**Figure 4:**
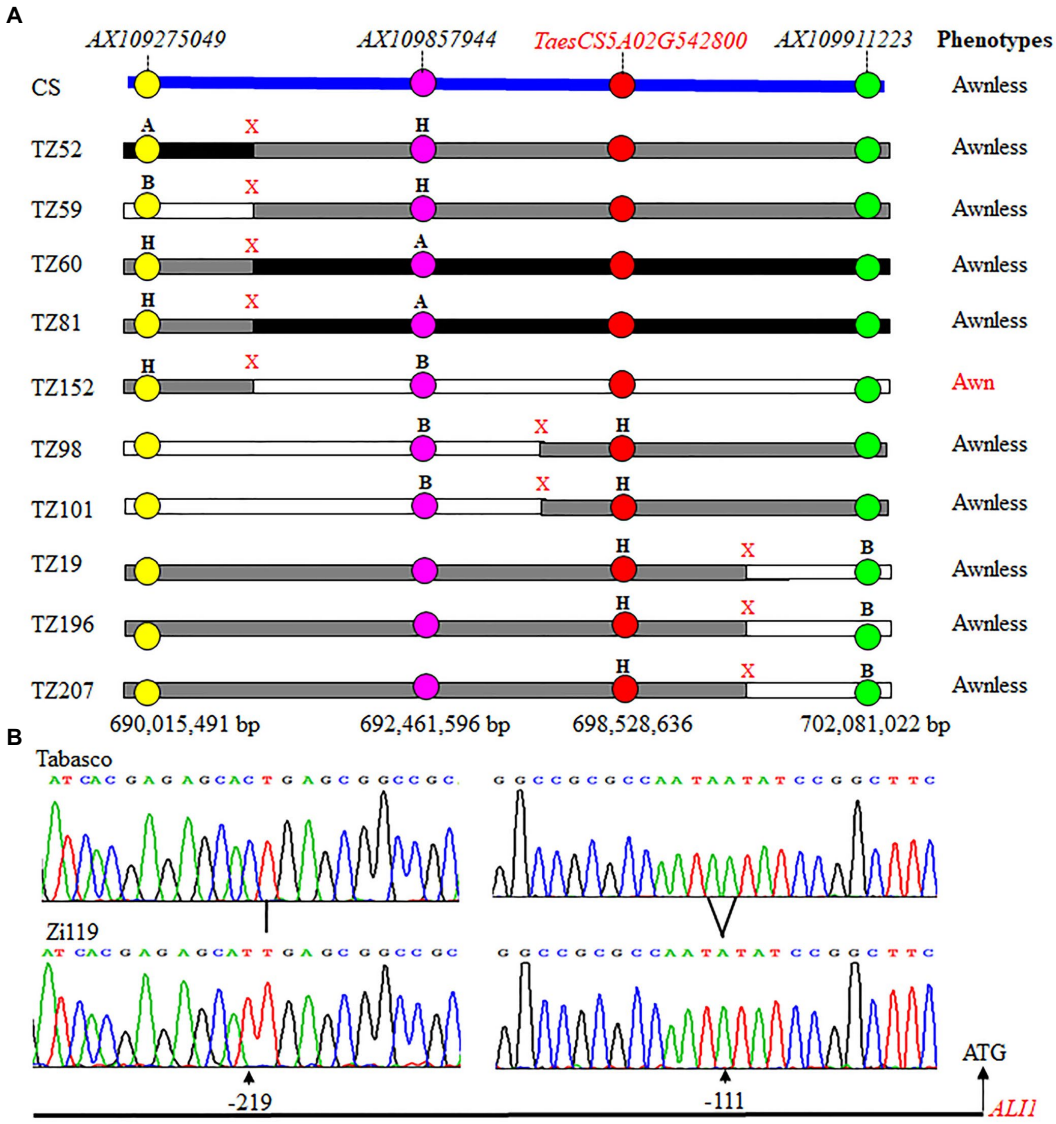
Genotypes and phenotypes of 10 recombinant plants that have crossovers at the *ALI-A1* locus. **(A)** Physical locations of 10 crossovers that occurred at the *ALI-A1* locus. Markers mapped chromosome arm 5AL that are indicated in different colors are arranged based on the sequences of Chinese Spring (CS). ALI-A1 is annotated *TaesCS5A02G542800*. “A” is the Tabasco allele, “B” for the Zi119 allele, and “H” for the heterozygote. “X” indicates a crossover detected between two neighboring markers. Phenotype of each recombinant plant was of awn or awnless. **(B)** Comparison of promoter sequences between the Tabasco allele and the Zi119 allele.

The Tabasco × Zi119 F_2_ population showed a clear cut-off segregation of awn and awnless spikes, fitting a 3: 1 (*χ*^2^ = 1.17, *p* = 0.28). The result demonstrated that the awn trait was dominant over the awnless trait in the F_2_ population. The integration of the phenotypes and genotypes for each of the F_2_ plants allowed mapping of a genomic region on chromosome 5A for this trait. Further analysis of SNP markers in the mapped genomic region identified 10 crossovers between *AX109275049* and *AX109911223* (Figure 4A). Fine mapping of these crossovers showed that the candidate gene should be located between *AX109857944* at 692,461,596 bp and *AX109911223* at 702,081,022 bp (Figure 4A). *TaesCS5A02G542800* representing *ALI-A1* at position 698,528,636 bp is located between the two SNP markers. TZ98 and TZ101 had homozygotes for *AX109857944* and heterozygotes for *AX109911223* and the two plants had no awn, indicating that the candidate gene was linked with *ALI-A1.* TZ19, TZ196, and TZ207 had homozygotes for *AX109911223* and heterozygotes for *AX109857944* and these three plants had no awn, indicating that the candidate gene was linked with *ALI-A1* too. Combining the genotypic and phenotypic results from these five critical recombinant plants, *ALI-A1* was a candidate gene causing the segregation of awn/awnless traits in the F_2_ population. Tabasco had the dominant *Ali-A1* allele for awn, Zi119 had the recessive *ali-A1* allele for awnless, and *ALI-A1* was used for the common allele.

### Allelic variation in *ALI-A1*

**Figure 5:**
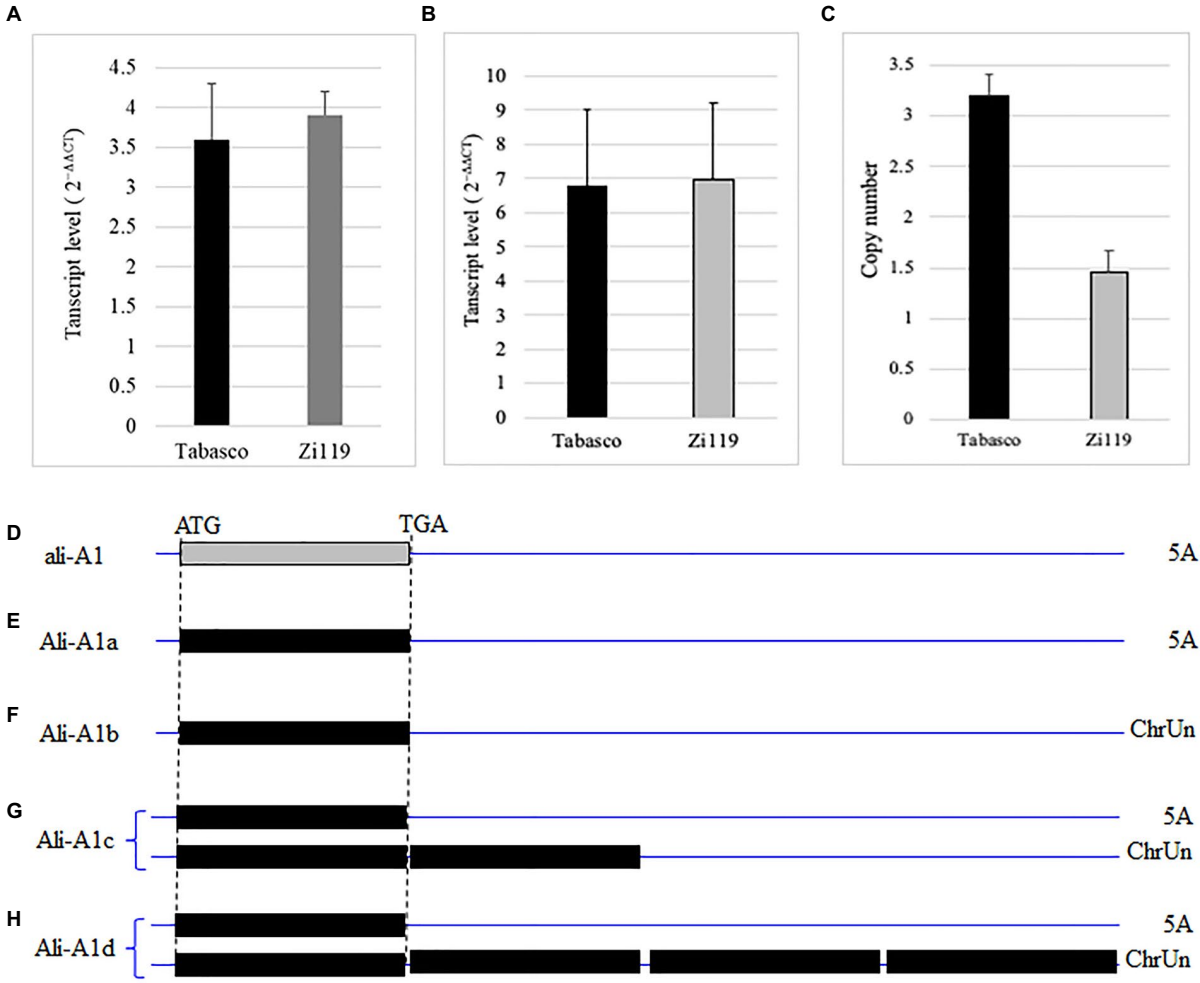
Transcript levels and copy number of different *ALI-A1* alleles. **(A)** Comparison of *ALI-A1* transcript levels in leaves between the Tabasco allele and the Zi119 allele. **(B)** Comparison of *ALI-A1* transcript levels in young spikes between the Tabasco allele and the Zi119 allele. **(C)** Comparison of *ALI-A1* copy number between the Tabasco allele and the Zi119 allele. **(D–H)** Copy number and chromosomal locations of ALI-A1 in CS and 10 wheat genome sequences. **(D)** The *Ali-A1* allele was found to have one copy on chromosome 5A in six cultivars including CS at position 700,824,508 bp, Landmark at position 700,026,149 bp, Jagger at position 700,709,285 bp, Lancer at position 692,663,330 bp, Mace at position 691,541,051 bp, and Norin at position 699,728,557 bp. **(E–H)** *Ali-A1* was found to have four types *Ali-A1a* through *Ali-A1d* based on their chromosomal locations. **(E)** *Ali-A1a* in Arina is at position 695,901,606 bp on chromosome 5A. **(F)** *Ali-A1b* in Stanley is at position 140,377,555 bp and Julius at position 114,994,011 on ChrUN (unknown chromosome). **(G)** *Ali-A1c* is in Mattis with three copies, one at position 688,692,384 bp on chromosome 5A, and another two copies at positions 239,812,783 and 287,868,636 bp on ChrUN. **(H)** *Ali-A1d* is in Spelta with five copies, one at position 698,375,770 bp on chromosome 5A and another four copies at positions 192,142,499, 192,159,103, 238,409,458, and 238,413,036 bp on ChrUN.

Sequencing results showed that the Tabasco and Zi119 *ALI-A1* alleles had the same sequences in the coding region but differed in two SNPs in the 254 bp promoter region (Figure 4B). One was at position-111, where “C” in the *Ali-A1* allele was replaced by “T” in the *ALI-A1* allele. The other one was at position-219, where “A” in the *Ali-A1* allele was deleted in the *ali-A1* allele. The transcript level of *ALI-A1* gene was determined and no significant difference was observed between the two alleles using RNA samples collected from leaves at the seedling stage (*p* = 0.666, *n* = 9; Figure 5A) or young spikes at heading date (*p* = 0.374, *n* = 9; Figure 5B).

The same primers were used to determine the copy number of genes. Relative to one copy of *ali-A1* in Zi119, *Ali-A1* in Tabasco was found to have two copies of *Ali-A1* (Figure 5C). According to the copy number and chromosomal locations of *ALI-A1* gene in CS and 10 wheat genome sequences, the *Ali-A1* allele was found to have one copy on chromosome 5A in six cultivars (Figure 5D). However, the *Ali-A1* allele was found to have four types, *Ali-A1a* through *Ali-A1d*. *Ali-A1a* has one copy on chromosome 5A (Figure 5E), *Ali-A1b* on has one copy ChrUN (unknown chromosome; Figure 5F), *Ali-A1c* has three copies, one at position on chromosome 5A and another two copies on ChrUN (Figure 5G), and *Ali-A1d* has five copies, one on chromosome 5A and another four copies on ChrUN (Figure 5H). It was likely that Tabasco has two copies of *ALI-A1*, one on chromosome 5A and the other on ChrUN, which is referred to as *Ali-A1e* allele.

## Discussion

Zi119 is a spring wheat cultivar that has no vernalization for flowering about 50–70 days (depending on experiment seasons in the greenhouse) as presented in this study. In wheat production, however, Zi119 is planted in October and harvested in next May, spanning approximate 7 months in field in Zone III; where a winter season is clear (Zhang et al., 2008). Zi119 wheat was found to have the *Vrn-D1* allele for early flowering, when this cultivar was crossed with strong winter cultivar Tabasco and the resulting F_2_ population was tested under constant warm temperature and long day conditions. In the field, the spring wheat cultivar is not accelerated but delayed for flowering by low temperature, whereas a winter cultivar is accelerated in the developmental transition for flowering (Li et al., 2013). The *Vrn-D1* allele could make spring wheat or semi-winter cultivar to flower in a difference of a few months in the greenhouse but a few days in the field.

The *Vrn1* gene was initially cloned from diploid wheat *T. monococcum* (Yan et al., 2003). The availability of the gene sequence allowed isolation of three homoeologous genes, *VRN-A1*, *VRN-B1*, and *VRN-D1* in common wheat and identification of allelic variations in each of them. Compared with *VRN-A1* that has multiple alleles (Yan et al., 2003, 2004a; Pidal et al., 2009; Golovnina et al., 2010; Li et al., 2013; Shcherban et al., 2015), *VRN-B1* and *VRN-D1* are observed to have fewer haplotypes. *Vrn-D1* in spring wheat has a large deletion in intron one in hexaploid wheat (Fu et al., 2005). The effect intensity of the three *VRN-1* genes was ranked as *Vrn-A1* > *Vrn-B1* > *Vrn-D1*, based on analyses on Triple Dirk isogenic lines (Loukoianov et al., 2005), Chinese wheat cultivars (Zhang et al., 2008), and a biparental population (Li et al., 2015). Due to the greatest effect of the *Vrn-A1a* allele, it has been incorporated into spring wheat cultivars in Canadian breeding programs and spring-sown wheat cultivars in China to provide frost avoidance in short-season environments or frozen damage in spring season (Iqbal et al., 2007; Zhang et al., 2008). This study provided experimental evidence for the desirable selection of *Vrn-D1* on ideal heading date in wheat Zone III. The genetic corporation of *Vrn-D1* with two genes on chromosome arms 2AS and 2DS could be used to make wheat cultivars with different developmental durations at joining state, heading date, and physiological maturity (Chen et al., 2009, 2010), which are adapted to gradually changed environments from southern to Northern regions in Zone III.

Awn Length inhibitor 1 (i.e., *TraesCS5A02G542800*) was believed to be a strong candidate for the dominant Tipped1 or B1 as an awn suppressor (DeWitt et al., 2020; Huang et al., 2020; Niu et al., 2020; Wang et al., 2020; Würschum et al., 2020). This gene was cloned by analyses of association maps and biparental populations, and polymorphisms were observed not in the *ALI1* coding region but a nearby region (a 30-bp deletion at 4 kb downstream) that was predictive of regulatory elements (DeWitt et al., 2020). It could be a 25-bp deletion upstream of the *Ali-1* allele that was linked with the awnless trait (Huang et al., 2020), but this deletion is also found in *T. urartu*, raising a doubt about the causal. It could be one of five SNPs at positions from-1,630 to-709 in the promoter region of *ALI1* that is responsible for awn elongation (Wang et al., 2020). In addition, SNPs at the ALI1 locus could be a gene that has a dosage effect on awn length based on segregation for long, intermediate and short awn phenotypes two F_8_ recombinant inbred lines (RILs; Niu et al., 2020). Collectively, it is not yet clear how allelic variation in *ALI-1* caused differences between the awnless and awn traits or between the long awn and short awn traits (Würschum et al., 2020). *ALI-1* is located within a genomic region, where it is surrounded by >100 kb of insertion or deletion of transposable or repetitive elements that may hinder assembly of genome sequences, resulting in unknown chromosome. Among the 10 sequenced genomes, *Ali-1* was found to have variation in gene copy number, which could result from duplication of chromosomal fragment. Determining if the observed duplication of chromosomal fragment containing *Ali-1* is a causative mechanism will require further work.

The presence of the two major genes in locally adapted cultivars provides easy selection in a short cycle. Such rapid cycling lines will not require vernalization to induce flowering and will be selected for traits of interest such as awn. The elite breeding lines will be crossed to the winter wheat recurrent parent used to create the rapid cycling lines to recover the winter growth habit, thus creating adapted backcross derived lines quickly and efficiently and accelerating winter wheat breeding schemes. Detection of vernalization genes by traditional genetic methods is time consuming. Fortunately, the cloned vernalization genes have facilitated the development of gene-specific markers or functional markers (also known as perfect or diagnostic markers). The markers provide a unique opportunity to screen large collections of wheat germplasm for allelic diversity at the desirable genes.

## Data availability statement

The original contributions presented in the study are included in the article/Supplementary material, further inquiries can be directed to the corresponding author.

## Author contributions

JW, LQ, YL, BF, RN, YR, and HJ conducted marker development, genotyping, and mapping. JW and LY conceived the project and designed the experiments. All authors analyzed data and reviewed the manuscript. JW, LQ, and LY wrote the manuscript. All authors contributed to the article and approved the submitted version.

## Funding

This study was partially funded by the National Key Research and Development Plan of China (2021YFD1200601-05) and the International Cooperation Fund of Jiangsu Academy of Agricultural Sciences. This project was supported by Agriculture and Food Research Initiative Competitive Grants (2017-67007-25939 and 2022-68013-36439) from the USDA National Institute of Food and Agriculture (NIFA).

## Conflict of interest

The authors declare that the research was conducted in the absence of any commercial or financial relationships that could be construed as a potential conflict of interest.

## Publisher’s note

All claims expressed in this article are solely those of the authors and do not necessarily represent those of their affiliated organizations, or those of the publisher, the editors and the reviewers. Any product that may be evaluated in this article, or claim that may be made by its manufacturer, is not guaranteed or endorsed by the publisher.

## Footnotes

^1^ https://urgi.versailles.inra.fr/

## Supplementary material

Supplementary Table S1 Detailed information for the length of each linkage group and genetic distances of the SNP markers on the whole genome.

## References

[ref1] Beales J. Turner A. Griffiths S. Snape J. W. Laurie D. A. (2007). A Pseudo-Response Regulator is misexpressed in the photoperiod insensitive Ppd-D1a mutant of wheat (*Triticum aestivum* L.). Theor. Appl. Genet. 115, 721–733. doi: 10.1007/s00122-007-0603-4, PMID: 17634915

[ref2] Chen Y. Carver B. Wang S. Cao S. Yan L. (2010). Genetic regulation of developmental phases in winter wheat. Mol. Breed. 26, 573–582. doi: 10.1007/s11032-010-9392-6, PMID: 24220102

[ref3] Chen Y. Carver B. F. Wang S. Zhang F. Yan L. (2009). Genetic loci associated with stem elongation and winter dormancy release in wheat. Theor. Appl. Genet. 118, 881–889. doi: 10.1007/s00122-008-0946-5, PMID: 19130033

[ref4] DeWitt N. Guedira M. Lauer E. Sarinelli M. Tyagi P. Fu D.. (2020). Sequence-based mapping identifies a candidate transcription repressor underlying awn suppression at the B1 locus in wheat. New Phytol. 225, 326–339. doi: 10.1111/nph.16152, PMID: 31465541 PMC6916393

[ref5] Dong Y. S. Zheng D. S. (2000). Wheat Genetic Resources in China. Beijing: China Agriculture Press

[ref260] Dubcovsky J. Galvez A. F. Dvorak J. (1994). Comparison of the genetic organization of the early salt stress response gene system in salt-tolerant Lophopyrum elongatum and salt-sensitive wheat. Theor. Appl. Genet. 87, 957–964. 24190530 10.1007/BF00225790

[ref6] Flood R. G. Halloran G. M. (1986). Genetics and physiology of vernalization response in wheat. Adv. Agron. 39, 87–125. doi: 10.1016/S0065-2113(08)60466-6

[ref7] Fu D. Szücs P. Yan L. Helguera M. Skinner J. S. Zitzewitz J. V.. (2005). Large deletions within the first intron in VRN-1 are associated with spring growth habit in barley and wheat. Mol. Gen. Genomics. 273, 54–65. doi: 10.1007/s00438-004-1095-4, PMID: 15690172

[ref8] Gao H. D. Zhu F. F. Jiang Y. J. Wu J. Z. Yan W. Zhang Q. F.. (2012). Genetic analysis and molecular mapping of a new powdery mildew resistant gene Pm46 in common wheat. Theor. Appl. Genet. 125, 967–973. doi: 10.1007/s00122-012-1886-7, PMID: 22660629

[ref9] Golovnina K. A. Kondratenko E. Y. Blinov A. G. Goncharov N. P. (2010). Molecular characterization of vernalization loci VRN1 in wild and cultivated wheats. BMC Plant Biol. 10, 168–182. doi: 10.1186/1471-2229-10-168, PMID: 20699006 PMC3095301

[ref10] Griffiths S. Dunford R. P. Coupland G. Laurie D. A. (2003). The evolution of CONSTANS-like gene families in barley, rice, and Arabidopsis. Plant Physiol. 131, 1855–1867. doi: 10.1104/pp.102.016188, PMID: 12692345 PMC166942

[ref11] Guo J. Zhang Y. Shi W. Zhang B. Zhang J. Xu Y.. (2015). Association analysis of grain-setting rates in apical and basal spikelets in bread wheat (*Triticum aestivum* L.). Front. Plant Sci. 6, 1029. doi: 10.3389/fpls.2015.01029, PMID: 26635852 PMC4653486

[ref13] Huang D. Zheng Q. Melchkart T. Bekkaoui Y. Konkin D. J. F. Kagale S.. (2020). Dominant inhibition of awn development by a putative zinc-finger transcriptional repressor expressed at the B1 locus in wheat. New Phytol. 225, 340–355. doi: 10.1111/nph.16154, PMID: 31469444 PMC6916588

[ref14] Iqbal M. Navabi A. Yang R. C. Salmon D. F. Spaner D. (2007). Molecular characterization of vernalization response genes in Canadian spring wheat. Genome 50, 511–516. doi: 10.1139/G07-028, PMID: 17612620

[ref15] Iwaki K. Nakagawa K. Kuno H. Kato K. (2000). Ecogeographical differentiation in East Asian wheat, revealed from the geographical variation of growth habit and Vrn genotype. Euphytica 111, 137–143. doi: 10.1023/A:1003862401570

[ref16] Jin S. B. (1997). Chinese Wheat Cultivars and Their Pedigrees (1983–1993). Beijing: China Agriculture Press

[ref17] Kato K. Yamagata H. (1988). Method for evaluation of chilling requirement and narrow-sense earliness of wheat cultivars. Jpn. J. Breed. 38, 172–186. doi: 10.1270/jsbbs1951.38.172

[ref18] Kippes N. Debernardi J. M. Vasquez-Gross H. A. Akpinar B. A. Budak H. Kato K.. (2015). Identification of the *VERNALIZATION 4* gene reveals the origin of spring growth habit in ancient wheats from South Asia. Proc. Natl. Acad. Sci. U. S. A. 112, E5401–E5410. doi: 10.1073/pnas.1514883112, PMID: 26324889 PMC4593092

[ref19] Laurie D. A. Pratchett N. Bezant J. H. Snape J. W. (1995). RFLP mapping of five major genes and eight quantitative trait loci controlling flowering time in a winter x spring barley (*Hordeum vulgare* L.) cross. Genome 38, 575–585. doi: 10.1139/g95-074, PMID: 18470191

[ref20] Li G. Wang Y. Chen M. S. Edae E. Poland J. Akhunov E.. (2015). Precisely mapping a major gene conferring resistance to *Hessian fly* in bread wheat using genotyping-by-sequencing. BMC Genomics 16, 108. doi: 10.1186/s12864-015-1297-7 25765046 PMC4347651

[ref21] Li G. Yu M. Fang T. Cao S. Carver B. F. Yan L. (2013). Vernalization requirement duration in winter wheat is controlled by *Ta*VRN-A1 at the protein level. Plant J. 76, 742–753. doi: 10.1111/tpj.12326, PMID: 24033823 PMC4282524

[ref22] Loukoianov A. Yan L. Blechl A. Sanchez A. Dubcovsky J. (2005). Regulation of *VRN-1* vernalization genes in normal and transgenic polyploid wheat. Plant Physiol. 138, 2364–2373. doi: 10.1104/pp.105.064287, PMID: 16055679 PMC1183422

[ref23] Niu J. Zheng S. Shi X. Si Y. Tian S. He Y.. (2020). Fine mapping and characterization of the awn inhibitor *B1* locus in common wheat (*Triticum aestivum* L.). Crop J. 8, 613–622. doi: 10.1016/j.cj.2019.12.005

[ref24] Pidal B. Yan L. Fu D. Zhang F. Tranquilli G. Dubcoveky J. (2009). The CArG-box located upstream from the transcriptional start of wheat vernalization gene *VRN1* is not necessary for the vernalization response. J. Hered. 100, 355–364. doi: 10.1093/jhered/esp002, PMID: 19251764

[ref25] Poland J. A. Rife T. W. (2012). Genotyping-by-sequencing for plant breeding and genetics. Plant Genome 5, 92–102. doi: 10.3835/plantgenome2012.05.0005

[ref26] Shcherban A. B. Strygina K. V. Salina E. A. (2015). *VRN-1* gene- associated prerequisites of spring growth habit in wild tetraploid wheat *T. dicoccoides* and the diploid A genome species. BMC Plant Biol. 15, 94. doi: 10.1186/s12870-015-0473-x 25888295 PMC4383061

[ref27] Snape J. W. Butterworth K. Whitechurch E. Worland A. J. (2001). Waiting for fine times: Genetics of flowering time in wheat. Euphytica 119, 185–190. doi: 10.1023/A:1017594422176

[ref28] Turner A. Beales J. Faure S. Dunford R. P. Laurie D. A. (2005). The pseudo-response regulator Ppd-H1 provides adaptation to photoperiod in barley. Science 310, 1031–1034. doi: 10.1126/science.1117619, PMID: 16284181

[ref29] Wang D. Yu K. Jin D. Sun L. Chu J. Wu W.. (2020). Natural variations in the promoter of *Awn Length Inhibitor 1* (*ALI-1*) are associated with awn elongation and grain length in common wheat. Plant J. 101, 1075–1090. doi: 10.1111/tpj.14575, PMID: 31628879

[ref30] Würschum T. Jähne F. Phillips A. L. Langer S. M. Friedrich C. Longin H.. (2020). Misexpression of a transcriptional repressor candidate provides a molecular mechanism for the suppression of awns by *Tipped 1* in wheat. J. Exp. Bot. 71, 3428–3436. doi: 10.1093/jxb/eraa106, PMID: 32103263 PMC7307850

[ref31] Yan L. Fu D. Li C. Blechl A. Tranquilli G. Bonafede M.. (2006). Wheat and barley vernalization gene *VRN3* is an orthologue of FT. Proc. Natl. Acad. Sci. U.S.A. 103, 19581–19586. doi: 10.1073/pnas.0607142103 17158798 PMC1748268

[ref32] Yan L. Helguera M. Kato K. Fukuyama S. Sherman J. Dubcovsky J. (2004a). Allelic variation at the *VRN-1* promoter region in polyploidy wheat. Theor. Appl. Genet. 109, 1677–1686. doi: 10.1007/s00122-004-1796-4 15480533

[ref33] Yan L. Loukoianov A. Tranquilli G. Blechl A. Khan I. A. Ramakrishna W.. (2004b). The wheat *VRN-2* gene is a flowering repressor down-regulated by vernalization. Science 303, 1640–1644. doi: 10.1126/science.1094305 15016992 PMC4737501

[ref34] Yan L. Loukoianov A. Tranquilli G. Helguera M. Fahima T. Dubcovsky J. (2003). Positional cloning of the wheat vernalization gene *VRN1*. Proc. Natl. Acad. Sci. U. S. A. 100, 6263–6268. doi: 10.1073/pnas.0937399100, PMID: 12730378 PMC156360

[ref35] Zhang X. K. Xia X. C. Xiao Y. G. Zhang Y. He Z. H. (2008). Allelic variation at the vernalization genes *Vrn-A1*, *Vrn-B1*, *Vrn-D1* and *Vrn-B3* in Chinese common wheat cultivars and their association with growth habit. Crop Sci. 48, 458–470. doi: 10.2135/cropsci2007.06.0355

[ref36] Zhuang Q. S. (2003). Wheat Improvement and Pedigree Analysis in Chinese Wheat Cultivars. Beijing: China Agriculture Press

